# γ-Secretase Modulatory Proteins: The Guiding Hand Behind the Running Scissors

**DOI:** 10.3389/fnagi.2020.614690

**Published:** 2020-12-02

**Authors:** Eitan Wong, Georgia R. Frost, Yue-Ming Li

**Affiliations:** Chemical Biology Program, Memorial Sloan Kettering Cancer Center, New York, NY, United States

**Keywords:** IFITM, hypoxia, neuroinflammation, neurodegeneration, enzyme

## Abstract

Described as the “proteasome of the membrane” or the “scissors in the membrane,” γ-secretase has notoriously complicated biology, and even after decades of research, the full extent of its regulatory mechanism remains unclear. γ-Secretase is an intramembrane aspartyl protease complex composed of four obligatory subunits: Nicastrin (NCT), Presenilin (PS), Presenilin Enhancer-2 (Pen-2), and Anterior pharynx-defective-1 (Aph-1). γ-Secretase cleaves numerous type 1 transmembrane substrates, with no apparent homology, and plays major roles in broad biological pathways such as development, neurogenesis, and cancer. Notch and the amyloid precursor protein (APP) and are undoubtedly the best-studied γ-secretase substrates because of their role in cancer and Alzheimer’s disease (AD) and therefore became the focus of increasing studies as an attractive therapeutic target. The regulation of γ-secretase is intricate and involves the function of multiple cellular entities. Recently, γ-secretase modulatory proteins (GSMPs), which are non-essential subunits and yet modulate γ-secretase activity and specificity, have emerged as an important component in guiding γ-secretase. GSMPs are responsive to cellular and environmental changes and therefore, provide another layer of regulation of γ-secretase. This type of enzymatic regulation allows for a rapid and fine-tuning of γ-secretase activity when appropriate signals appear enabling a temporal level of regulation. In this review article, we discuss the latest developments on GSMPs and implications on the development of effective therapeutics for γ-secretase-associated diseases such as AD and cancer.

## γ-Secretase and Alzheimer Disease (AD)

Alzheimer’s disease (AD) is the most common neurodegenerative disease manifested in dementia symptoms that gradually worsen with age. AD pathology is characterized by the presence of extracellular plaques and intracellular neurofibrillary tangles in the brain. According to the amyloid cascade hypothesis (Hardy and Higgins, 1992; Hardy and Selkoe, 2002), β-amyloid peptide (Aβ), the major constituent of plaques, is the causative mediator driving the deterioration and neuropathology of AD. The Aβ peptides are derived from amyloid precursor protein (APP) after processing by two proteases, first, β-secretase (BACE1) producing the β-C-terminal fragment (βCTF), and second, γ-secretase cleavage, creating an amyloid intracellular domain (AICD) and Aβ of different lengths (Crump et al., 2013).

AD can be classified into two major types: familial AD (FAD) and sporadic AD (SAD; Bateman et al., 2011). The pathological features and functional connectivity of both forms of AD are similar (Bateman et al., 2013; Thomas et al., 2014), suggesting that FAD can be an effective model to study the pathogenic mechanism of SAD. Mutations in one of three genes, *APP*, *presenilin-1 (*PSEN1), and *presenilin-2 (*PSEN2), or duplication of *APP* cause FAD (Goate et al., 1991; Levy-Lahad et al., 1995; Sherrington et al., 1995). Presenilin-1 (PS1) and presenilin-2 (PS2) are polytopic membrane proteins with nine transmembrane domains that are synthesized as a single polypeptide (Spasic et al., 2006). Both the PS1 and PS2 proteins are endoproteolyzed, whereby the N- and C-terminal cleavage products (NTF and CTF) remain associated in the heterodimeric form (Thinakaran et al., 1996).

The discovery of PS has provided critical insight into the identity of γ-secretase. (1) FAD mutations in *PSEN1* and *PSEN2* increase in the ratio of Aβ42 to Aβ40 in transfected cells and in transgenic mice (Borchelt et al., 1996; Duff et al., 1996; Scheuner et al., 1996; De Strooper, 2007; Wolfe, 2007). (2) Cultured neurons isolated from PS1-deficient mice produce significantly less Aβ and APP fragments that are not processed by γ-secretase is being accumulated (Naruse et al., 1998; De Strooper et al., 1998). (3) γ-Secretase activity is abolished in cells cultured from PS1 and PS2 deficient mice (Herreman et al., 2000; Zhang et al., 2000). (4) γ-Secretase activity is reduced by mutagenesis of two conserved aspartate residues in the transmembrane regions of PS1 (Wolfe et al., 1999). (5) γ-Secretase activity is connected to PS-containing macromolecular complexes (Li et al., 2000a). (6) Active-site directed γ-secretase inhibitors directly bind to PS1 or PS2 (Esler et al., 2000; Li et al., 2000b; Xu et al., 2002). (7) A recombinant PS1 variant alone in the purified reconstitution system has γ-secretase activity, offering the final proof that PS is γ-secretase (Ahn et al., 2010).

## γ-Secretase Essential Subunits and Basic Regulation

A broad range of γ-secretase substrates has been identified (Haapasalo and Kovacs, 2011), indicating the diverse biological functions of this protease. The most notable substrate of γ-secretase is APP, the precursor for Aβ peptides, which aggregates to form the core of senile plaque in AD patients (Guo et al., 2020). Another critical substrate which has been extensively investigated is Notch, a cell surface protein, which participates in cell-cell contact signaling and is essential for propriate embryonic development (De Strooper et al., 1999). Notch plays an important role in different types of cancer (Lobry et al., 2011), therefore, γ-secretase is an appealing drug target in both AD and cancer. However, because of the wide spectrum of γ-secretase substrates, it is extremely difficult to develop selective inhibitors, as evidenced by the failure of γ-secretase inhibitors in clinical trials due to side effects from non-selective inhibition (Haapasalo and Kovacs, 2011).

It is known that γ-secretase is an intramembrane protease dependent on the assembly of four subunits: Nicastrin (NCT), PS, Presenilin Enhancer-2 (Pen-2), and Anterior pharynx-defective-1 (Aph-1; De Strooper et al., 2012; Crump et al., 2013). Structures of human γ-secretase complex alone or with substrates obtained by cryo-electron microscopy (cryo-EM), have offered novel insights into the structural basis of function and recognition as well as the flexibility and complexity of this enzyme (Lu et al., 2014; Sun et al., 2015; Yang et al., 2019; Zhou et al., 2019). Also, the endogenous γ-secretase complexes appear to range in size with different levels of activity (Gu et al., 2004; Evin et al., 2005), suggesting that different subunit stoichiometry and cofactors composition might contribute to activity and substrate specificity (Placanica et al., 2010). The mechanisms by which γ-secretase is being regulated at the cellular level have been the focus of extensive studies that uncover in part its intricate biology.

The regulation of γ-secretase starts with the synthesis and assembly of the complex. The correct assembly of the γ-secretase complex is tightly regulated and requires multiple cellular events to generate the mature and active form (Takasugi et al., 2003; Kim et al., 2004). While the basic functional γ-secretase complex requires at least one of each of its essential subunits (Sato et al., 2007), the existence of PS and Aph-1 isoforms (PS1/PS2 and Aph-1a/Aph-1b), as well as splice variants (Aph-1aS and Aph-1aL), introduces further permutations to the basic complex (Lai et al., 2003; Shirotani et al., 2004). Furthermore, different variations of the γ-secretase complex can exist in the same tissue and even in the same cells (Placanica et al., 2009a, b).

Compartmentalization between γ-secretase and its different substrates in distinct cellular domains likely plays a role in γ-secretase substrate availability. Rather than altering γ-secretase activity to favor one substrate over another, spatial separation offers a post-translational regulation mechanism. For example, APP is processed by intracellular γ-secretase, while notch, which functions in the plasma membrane, is processed at the cell surface (Tarassishin et al., 2004). Multiple lines of evidence also suggest that lipid composition and lipid rafts which simulate the different cellular compartments directly affect the cleavage and distribution of γ-secretase. Changes to lipid composition in the membrane also impact substrate processing (Osenkowski et al., 2008) and in particular cholesterol-rich membranes are the major site of Aβ production (Wahrle et al., 2002; Marquer et al., 2014). Interestingly, a recent report suggests that not only are APP and Notch processing modulated by membrane lateral organization but also that γ-secretase can actively recruit specific membrane components that create a lipid environment favorable for substrate recognition and activity (Barros et al., 2020).

## The Context-Dependent γ-Secretase Modulatory Proteins (GSMPs)

The regulation of γ-secretase has been and continues to be, a major question in the field because of its numerous substrates that regulate many biological processes ranging from neuronal development, angiogenesis to tumorigenesis (Jurisch-Yaksi et al., 2013). While the expression of the essential γ-secretase components is ubiquitous in all tissues, only a small percentage of γ-secretase complexes are catalytically active (Lai et al., 2003). The existence of the inactive complex raises three critical questions: (1) Why does the inactive complex exist? (2) Can the inactive complex be activated? and (3) Does the activation play a role in AD and cancer? Furthermore, since the gene expression and the protein level of PS are not correlated with enzymatic activity in cells, it is challenging to assess the active γ-secretase complex (Lai et al., 2003; Placanica et al., 2009a). Therefore, the development of accurate γ-secretase activity assays (Shelton et al., 2009a, b), as well as photoaffinity labeling using a transition state inhibitor, are valuable tools to study γ-secretase (Crump et al., 2013; Nie et al., 2020).

**Figure 1:**
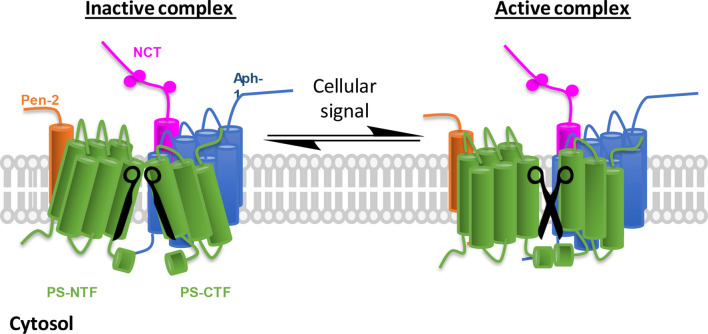
γ-Secretase complex activation in response to cellular environmental changes. Schematic representation of inactive/active γ-secretase equilibrium. γ-Secretase obligatory subunits are abundantly expressed in all tissues and cells, however, most of the γ-secretase complexes are not catalytically active (left, the two blades of scissors are not at correct position). To adapt cellular changes, γ-secretase can be activated by turning the inactive complexes to active ones (right, functional scissors) that selectively promote the cleavage of a specific substrate. The implication is 2-fold: first, inactive γ-secretase complexes are physiologically important, and second, γ-secretase can be temporally regulated.

The notion that an abundant protease like γ-secretase must respond to changes in the environment has led to the hypothesis that a fraction of the γ-secretase complexes exist in a partially dormant state and can be activated and attenuated by binding of γ-secretase modulatory proteins (GSMPs; Figure 1). Numerous proteins had been identified to interact with the core complex and impact γ-secretase activity (Chen et al., 2006; Vetrivel et al., 2007; Gertsik et al., 2014; Villa et al., 2014; Wong et al., 2019; Hur et al., 2020; Jung et al., 2020). In this review article, we will focus on the recent progress GSMPs and their regulation.

## The Missing Link Between Neuroinflammation and γ-Secretase in AD

Although genetic and animal studies support that Aβ peptides play a causative role in AD, the physiological role of Aβ is not clear. Recent studies suggest that Aβ peptides have antimicrobial and antiviral properties and function as part of the innate immune response of the brain (Vijaya Kumar et al., 2016; Eimer et al., 2018). Also, neuroinflammation has been established as a critical component of AD pathogenesis (Heneka et al., 2015), therefore, a connection between the two elements is under active investigation. Recently, a molecular link between innate immunity and AD was reported, interferon-induced transmembrane protein 3 (IFITM3) binds to γ-secretase and intensifies Aβ plaque formation (Hur et al., 2020).

IFITM3 is an innate immune response protein known to be involved in the cellular response to viral infections (Bailey et al., 2014) by inhibiting the entry of viruses to the host cells (Amini-Bavil-Olyaee et al., 2013). IFITM3 is highly upregulated by pro-inflammatory cytokines including both types I and II Interferon. Although IFITM3 was suggested to be a binding partner for γ-secretase, the underlying mechanism of interaction remained unclear (Wakabayashi et al., 2009). Recently, it was demonstrated that not only does IFITM3 directly bind to the γ-secretase complex, but it also upregulates γ-secretase activity for Aβ production. This means that IFITM3, in addition to its role in viral entry restriction, may trigger the production of anti-microbial peptide Aβ as an innate immune response by increasing γ-secretase activity. Interestingly, the binding of IFTIM3 to γ-secretase reduces Notch cleavage. This unprecedented property of IFITM3 to upregulated Aβ as an innate immune response may be a double-edged sword as it also contributes to the accumulation of Aβ in the brain. IFITM3 knockout in the AD mouse model (5XFAD) significantly reduces plaque deposition (Hur et al., 2020). This finding provides mechanistic evidence that immune activation contributes to the production of Aβ by increasing γ-secretase activity during infection and inflammation.

## Risk Factors and Sporadic AD (SAD)

Though rare, FAD mutations have provided critical insights into the underlying mechanisms of AD through the involvement of APP and γ-secretase, however, the causes for SAD are poorly understood. SAD is a complex and heterogeneous neurodegenerative disease attributed to multiple factors including genetics, aging, lifestyle, and environment. Apolipoprotein E4 (APOE4) is a major risk factor for SAD in an isoform-dependent manner influencing susceptibility for ~50% of cases (Corder et al., 1993; Bu, 2009). A recent study shows that *Apoe^−/−^* transgenic mice recapitulate transcriptomic signatures of human SAD samples (Pandey et al., 2019). Gender impacts the APOE effect, as females have almost a 2-fold greater risk of AD compared to males (Chene et al., 2015), which might be associated with the gut microbiome (Maldonado Weng et al., 2019). It is thought that APOE influences AD risk through regulating the inflammatory response and Aβ aggregation and clearance (Bales et al., 2009; Keren-Shaul et al., 2017; Krasemann et al., 2017; Liu et al., 2017). However, recent studies have shown that peripheral APOE has no apparent effect on Aβ accumulation in APP/PS1 transgenic AD mouse model (Huynh et al., 2019). Furthermore, APOE isoforms affect the expression of APP and the production of Aβ in neuronal cells (Huang et al., 2017; Wang et al., 2018). Whether APOE regulates γ-secretase in neurons remains to be investigated. Also, it has been reported that APOE4 affects Tau-mediated neurodegeneration (Shi et al., 2017). APOE4 contributes to the pathogenesis of AD through multiple pathways and is an appareling target for drug development (Liu et al., 2013).

Genome-wide association studies and identification of rare variants associated with AD have highlighted genes that are potential risk factors for AD (Karch and Goate, 2015). Triggering receptor expressed on myeloid cells 2 (TREM2) and CD33 are both expressed in myeloid-linage cells including microglia are associated with AD (Bertram et al., 2008; Guerreiro et al., 2013; Jonsson et al., 2013). Both TREM2 and CD33 regulate microglia-mediated uptake and clearance of Aβ (Griciuc et al., 2013; Wang et al., 2015; Ulrich and Holtzman, 2016). TREM2 was shown to directly bind to Aβ oligomers, activating the TREM2-dependent signaling pathway, and promotes microglial migration *in vitro* and clustering *in vivo* (Zhao et al., 2018; Zhong et al., 2018). While a loss of function variant of TREM2 (R47H), which is linked with a high risk of SAD, exhibits impairment in microglia associated plaque in an AD mouse model with knock-in TREM2 (R47H; Cheng-Hathaway et al., 2018). The role of TREM2 which is strongly associated with the risk of developing AD, confirms the important part of microglia and innate immune system in AD pathogenesis (Gratuze et al., 2018).

The identification and profiling of Disease-Associated Microglia (DAM) have further deepened our understanding of the contribution of innate immunity to AD (Keren-Shaul et al., 2017). Markers of inflammation are upregulated in AD mouse models and AD patients (Patel et al., 2005; Taylor et al., 2014) and peripheral myeloid cells in patients of different AD stages have increased pro-inflammatory profile correlated with disease progression (Thome et al., 2018). Proteomic profiling of microglia from the AD mouse model (5×FAD) identified similar pro-inflammatory changes as have been observed in LPS-treated mice (Rangaraju et al., 2018). Collectively, this suggests that AD pathology presents characteristics of chronic inflammatory disease. Interestingly, aging, the biggest risk factor for AD, has been shown to induce type I IFNs that modulate brain function (Baruch et al., 2014) and IFITM3 was found to be increased in a subset of SAD patients postmortem samples and in aged mice (Hur et al., 2020). Additionally, the strong correlation between the amount IFITM3 associated with γ-secretase and enzymatic activity for Aβ cleavage indicates that IFITM3 protein could be a marker of γ-secretase activity in SAD (Hur et al., 2020). Moreover, TREM2 is a substrate of γ-secretase (Wunderlich et al., 2013) and how GMSPs, such as IFITM3, affect the processing of TREM2 and the function of microglia remains to be investigated.

## The Modulation of γ-Secretase Under Hypoxic Condition

Hypoxia is a condition in which cells or tissues are deprived of adequate oxygen supply which triggers a cellular response orchestrated by the master transcriptional regulator Hypoxia-inducible factor 1-α (Hif-1α; Mukherjee et al., 2011). Hif-1α is continuously expressed but rapidly degrades in normal oxygen conditions (Jaakkola et al., 2001), whereas in hypoxic conditions Hif-1α is stabilized and translocates to the nucleus. In the nucleus, Hif-1α binds to and activates genes that are involved with vasomotor control, cell proliferation, angiogenesis, and cell metabolism (Maxwell et al., 1999). Surprisingly, a non-canonical role of Hif-1α was revealed as a GMSP. Under hypoxic conditions, Hif-1α can directly bind to the γ-secretase complex and upregulates Notch cleavage activity (Villa et al., 2014). It was shown that hypoxia increases γ-secretase cleavage of Notch, and this is dependent on Hif-1α. Interestingly, in a breast cancer model, the primary tumor grows into a proportion which generates hypoxic regions that stabilize Hif-1α. In turn, Hif-1α binds to and modulates γ-secretase activity which promotes migration and metastasis through activation of Notch downstream genes. It was suggested that Hif-1α can shift the equilibrium from inactive to active γ-secretase complexes, in response to low oxygen (Villa et al., 2014). Thus, increased γ-secretase activity and Notch signaling are essential for hypoxia-induced cell migration, invasion, and metastasis of breast cancer. This study provided the first evidence for temporal activation of γ-secretase complexes caused by changes in the cellular environment. This type of enzymatic regulation allows for a rapid switch from “off” state to “on” state when an appropriate signal appears enabling a temporal level of regulation.

## γ-Secretase Activating Protein (GSAP)

Imatinib (Gleevec), a drug for cancer treatment, was found to be selectively inhibiting Aβ secretion with no effect on Notch processing (Netzer et al., 2003). To identify Imatinib target protein, a photoactivatable form was synthesized and was found to specifically label an orphan protein, namely γ-secretase activating protein (GSAP; He et al., 2010). Knockdown of GSAP in an AD model transgenic mice (APPsweXPS1ΔE9) reduces Aβ burden (He et al., 2010) and treatment with imatinib, which binds to GSAP, reduces GSAP expression, Aβ levels, and tau phosphorylation in another AD mouse model (3xTg; Chu et al., 2014). It was hypothesized that GSAP simultaneously interacts with APP βCTF and γ-secretase, increasing Aβ generation (He et al., 2010). However, the following study brought the mechanism of GSAP in γ-secretase regulation into question, reporting that while knockdown of GSAP reduces Aβ production in cells, it is not through the interaction with βCTF (Hussain et al., 2013). A later study revealed that GSAP modulates γ-secretase activity by altering the active site and subsequently enhancing APP processing while not affecting Notch processing (Wong et al., 2019). In the case of GSAP, the precise cellular cue that is responsible for its interaction with γ-secretase is unclear, but there is an SNP in GSAP that is associated with AD, which might associate GSAP as a disease-related risk factor (Zhu et al., 2014; Perez et al., 2017). This was the first study that demonstrated the extent to which GSMPs attenuate γ-secretase activity by interacting with the enzyme in the allosteric site and changing its active site conformation.

## Serp1 Functions as the GSMP in Metabolic Stress Conditions

Diabetes is one of many risk factors associated with a greater risk of dementia diseases including AD (Ninomiya, 2014). High glucose levels, like in diabetes, induce endoplasmic reticulum (ER) stress and directly enhance Aβ production both *in vitro* and *in vivo* (Zhong et al., 2012; Nagai et al., 2016; Tharp et al., 2016). Stress-associated ER protein 1 (SERP1) is a small protein that interacts with and protects unfolded target proteins against degradation and is upregulated during ER stress. It was found that SERP1 stabilizes the APH1A/NCT subcomplex and promotes the localization of the γ-secretase complex in lipid raft (Jung et al., 2020). Subsequently, SERP1 facilitates the spatial distribution of γ-secretase resulting in increased APP processing in lipid rafts, the suggested site for APP cleavage (Wahrle et al., 2002; Osenkowski et al., 2008), while having virtually no impact on Notch cleavage. Postmortem AD samples have increased SERP1 expression and knocking down SERP1 in cells and mouse hippocampus decreases Aβ production. This study reveals the modulation of γ-secretase activity under ER stress enabled by the SERP1 acting as GSMP.

## Conclusions

**Figure 2:**
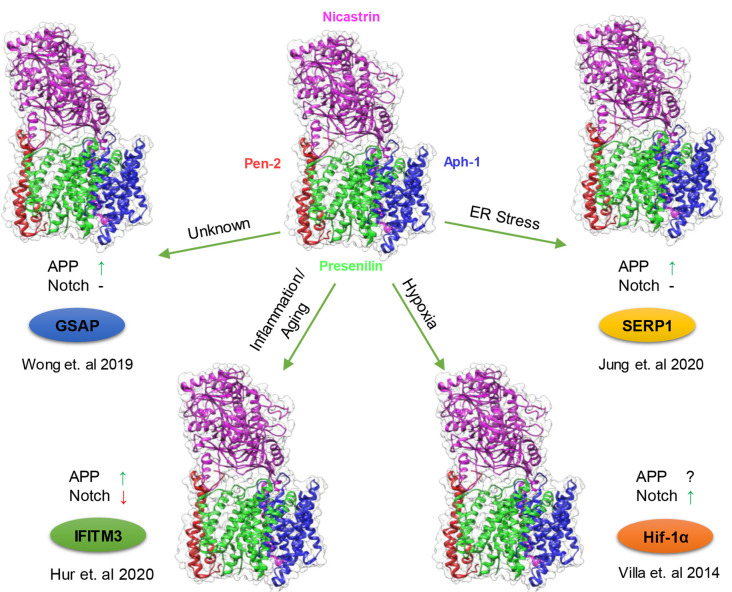
Modulation of γ-secretase by γ-secretase modulatory proteins (GSMPs). Schematic representation of the conditions in which GSMP triggers the attenuation of γ-secretase activity and selectivity. Cells possess a different type of γ-secretase complexes with variable levels of activities (PDB structure: 6IDF), which under certain cellular circumstances GSMP can bind and modify its activity and selectivity (clockwise): endoplasmic reticulum (ER) stress upregulate stress-associated ER protein 1 (SERP1) expression which binds and localize the γ-secretase complex to lipid rafts where amyloid precursor protein (APP) resides thus increasing APP cleavage but not Notch processing (Jung et al., 2020), hypoxic conditions stabilize Hif-1α which in turn binds to γ-secretase and increase its activity for Notch substrates (Villa et al., 2014), innate immune response and aging triggers the binding of interferon-induced transmembrane protein 3 (IFITM3) thus enhancing γ-secretase for APP but reducing Notch processing (Hur et al., 2020) and under unknown conditions GSAP attenuates γ-secretase activity solely towards APP but not Notch (Wong et al., 2019; Magenta—Nicastrin, Green—Presenilin, Blue—Aph-1 and Red—Pen-2).

**Figure 3:**
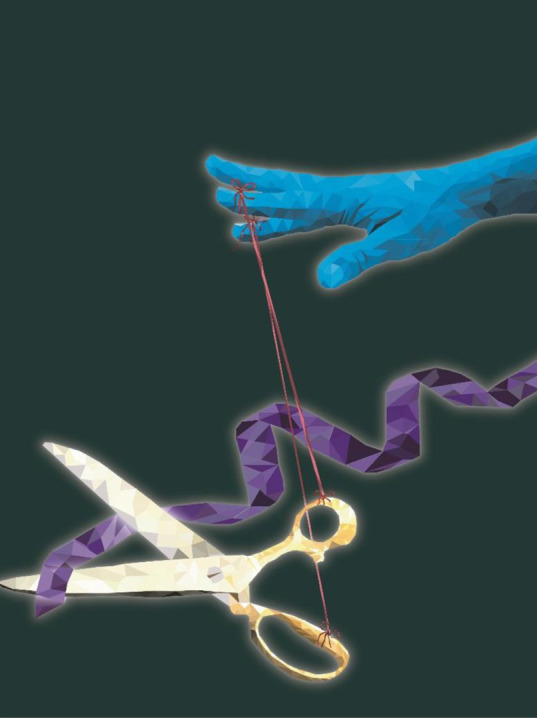
GSMPs pull the strings of γ-secretase activity. GSMPs act as a guiding hand to modulate γ-secretase activity in response to cellular and environmental cues such as hypoxia, inflammation, ER stress, and aging.

From the initial discoveries that mutations in PS1 and PS2 cause FAD and that PS1 and PS2 are the catalytic subunit of γ-secretase, extensive research on γ-secretase has revealed an overwhelming number of substrates of this protease and its involvement in broad aspects of biological processes. At first glance, γ-secretase may seem like a promiscuous enzyme earning its moniker as the “scissors in the membrane” its regulation is as complex and as variable as its involvement in multiple signaling pathways. The scientific community has come a long way in understanding these regulatory mechanisms from the assembly of essential subunits, complex formation to lipid composition. Yet, the discoveries of GMSPs present an interesting mode of regulation, adding another layer to the already complicated biology of γ-secretase. These nonessential subunits can fine-tune γ-secretase activity and specificity upon the appropriate cellular signal. It makes intuitive sense that an omnipresence protease like γ-secretase, which participates in numerous pathways, requires a quick attenuation when a signal appears to trigger the proper cellular response. Cells possess multiple γ-secretase complexes with different levels of activities, in which the majority are inactive and can serve as a pool by which a GSMP can bind and rapidly “activate”. Recent studies demonstrate that multiple GSMP can be recruited to modify γ-secretase activity and specificity under certain circumstances: IFITM3 induced by innate immune response and aging, Hif-1α stabilized by hypoxic condition, GSAP supposedly elevated in aging and AD and SERP1 under ER stress (Figure 2). Interestingly, each modulator has a different effect on γ-secretase substrates selectivity, indicating the wide range of impact on γ-secretase by GSMPs. The multiple γ-secretase-dependent signaling pathways suggest that more GMSPs might exist which modulates γ-secretase in response to other environmental changes. Thus, metaphorically, GMSPs may be the guiding hands behind “the running scissors” (Figure 3).

The molecular understanding of this type of regulation can be harnessed for therapeutic intervention. Although small-molecule γ-secretase modulators are still being developed to selectively inhibit γ-secretase activity for APP, while sparing Notch, as potential treatment (Mekala et al., 2020), it is worthwhile to also explore this new avenue and target GSMPs. Developing therapeutics to prevent the engagement of transient and context-dependent interactions with GSMP, instead of directly targeting γ-secretase, could reduce potential side effects. Thus, this emerging level of γ-secretase regulation may enable the development of targeted therapies for AD and cancer.

## Author Contributions

All authors prepared the manuscript and figures. All authors contributed to the article and approved the submitted version.

## Conflict of Interest

YM-L is a co-inventor of the intellectual property (assay for gamma secretase activity and screening method for gamma secretase inhibitors) owned by MSKCC and licensed to Jiangsu Continental Medical Development. The remaining authors declare that the research was conducted in the absence of any commercial or financial relationships that could be construed as a potential conflict of interest.
